# Diagnostic Potential of Cytokine Biomarkers in Endometriosis: Challenges and Insights

**DOI:** 10.3390/biomedicines12122867

**Published:** 2024-12-17

**Authors:** Laura Krygere, Povilas Jukna, Kristina Jariene, Egle Drejeriene

**Affiliations:** 1 Department of Obstetrics and Gynaecology, Medical Academy, Lithuanian University of Health Sciences, LT-50161 Kaunas, Lithuania; laura.kygere@lsmu.lt (L.K.); povilas.jukna@lsmu.lt (P.J.); kristina.jariene@lsmu.lt (K.J.); 2 Department of Obstetrics and Gynaecology, Hospital of Lithuanian University of Health Sciences Kauno Klinikos, LT-50161 Kaunas, Lithuania

**Keywords:** endometriosis, cytokines, diagnostics, biomarkers, gene polymorphism

## Abstract

Endometriosis is a common gynecological condition affecting approximately 10% of women of reproductive age, characterized by the abnormal presence of endometrial-like tissue outside the uterus. Although endometriosis was first described over 300 years ago, its underlying mechanisms remain poorly understood, and accurate, prompt diagnosis continues to be challenging. Currently, there is a lack of effective, non-invasive diagnostic methods, and available treatments often come with significant side effects and high recurrence rates. This has spurred interest in investigating the role of pro- and anti-inflammatory molecules, particularly cytokines, in endometriosis, as these molecules play a key role in its progression by influencing cell growth and differentiation. Previous studies suggest that various cytokines could serve as potential biomarkers for diagnosing endometriosis, as they are detectable in both serum and peritoneal fluid. This review provides an overview of the expression, origin, function, and regulation of specific cytokines in endometriosis, along with a brief discussion on their potential clinical implications for diagnosis. Due to the complexity of endometriosis, a panel of multiple biomarkers may ultimately be necessary for accurate diagnosis. It is essential to consider factors such as patient selection, sample collection, and analytical variability when initiating or evaluating biomarker studies.

## 1. Introduction

Endometriosis is a prevalent gynecological disorder, affecting approximately 10% of women of reproductive age, characterized by the ectopic presence of endometrial-like tissue outside the uterine cavity [1]. The majority of endometriosis cases occur in women between menarche and menopause, with the highest incidence typically seen between the ages of 25 and 45. Endometriosis was first described by Daniel Shroen in 1690, with its symptoms outlined by Arthur Duff in 1769. The condition’s pathogenesis began appearing in the literature in the late 19th century, and in 1860, Karl von Rokitansky defined it as the presence of endometrial tissue outside the uterine cavity [2]. The exact mechanisms of endometriosis are still not fully understood. One widely accepted explanation is the theory proposed by Sampson, known as “retrograde menstruation”. This theory suggests that endometriosis develops when menstrual blood is pushed back into the peritoneal cavity through the fallopian tubes, leading to the formation of endometrial tissue outside the uterus [3,4]. However, the literature suggests that 90% of women with open fallopian tubes experience backward flow of menstrual blood and only 10% develop endometriosis. This indicates that other factors, including genetic predisposition and immune system dysfunctions, play a role in the survival and implantation of endometrial cells in the peritoneal cavity [2]. Endometriosis is also recognized as a chronic inflammatory condition linked to immune system processes [5]. Immune system dysfunction is present at nearly every stage of the disease’s progression. Macrophages, which play a crucial role in identifying foreign cells and presenting them to T cells, are more abundant in the peritoneal cavity of women with endometriosis but exhibit reduced phagocytic activity [6]. Macrophages secrete elevated levels of pro-inflammatory cytokines. The enhanced release of pro-inflammatory cytokines, combined with the reduced production of anti-inflammatory factors from stromal, epithelial, smooth muscle, or immune cells, plays a crucial role in the initiation, progression, and development of endometriosis [2,7]. Genetic factors, particularly gene polymorphisms, significantly influence the immune response by altering cytokine production and function [8]. These genetic variations can affect disease susceptibility, progression, and response to treatment [6,8].

A thorough medical history, gynecological examination with a speculum, bimanual examination, and additional diagnostic tests such as imaging techniques, laparoscopy, and biochemical tests are valuable in the initial diagnosis of the condition [2,9]. Endometriosis is associated with chronic pelvic pain, dysmenorrhea, deep dyspareunia, infertility, and pelvic organ dysfunction [1]. Unfortunately, there is no reliable serum marker for endometriosis, and imaging often fails to detect the full extent of the disease. Ultrasound is effective for diagnosing endometriomas with 83% sensitivity and 89% specificity, but for deep-infiltrating endometriosis affecting areas like the uterosacral ligaments and bladder, the sensitivity and specificity of transvaginal ultrasound range from 53% to 93%. Recently, high-resolution magnetic resonance imaging with bladder, vaginal, and rectal contrast has been a significant advancement. Laparoscopy is the preferred method for diagnosing endometriosis, allowing for histological confirmation through surgical biopsy, and should ideally be performed by experienced surgeons [9]. Recently, microRNAs (miRNAs) have shown promising results in the diagnostics of endometriosis due to their stability in biological fluids and tissue specificity. Although no single miRNA or panel has yet achieved the required specificity and sensitivity, early studies suggest encouraging potential for miRNAs as noninvasive biomarkers for diagnosing endometriosis [10].

**Figure 1:**
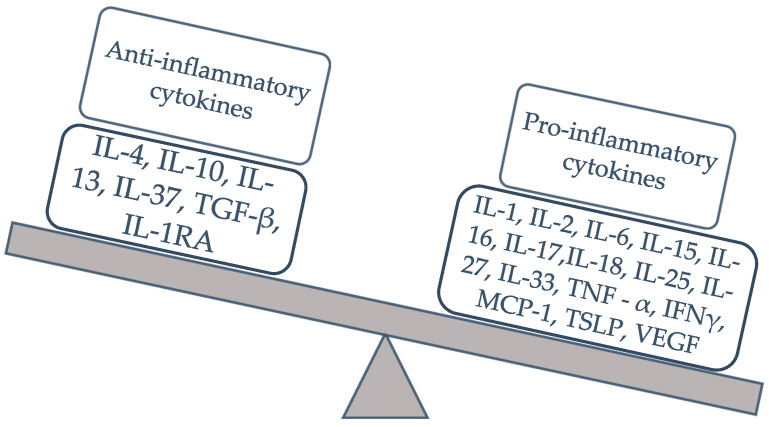
The balance of pro-inflammatory and anti-inflammatory cytokines in endometriosis.

Despite advancements in diagnostic techniques and a deeper understanding of endometriosis pathophysiology, patients often face significant clinical challenges due to the limitations of non-invasive diagnostic methods and the suboptimal efficacy of current treatments, which are frequently accompanied by adverse side effects and a high rate of recurrence. As a result, endometriosis severely diminishes patients’ quality of life, impacting their social and family relationships and contributing to substantial healthcare costs [1,2,11]. Given the significant role of inflammatory immune responses in the development and progression of endometriosis, researchers have explored cytokines as potential non-invasive serum biomarkers for diagnosis and as treatment targets [1]. Cytokines are proteins with diverse biological functions, including roles in cell proliferation and differentiation. Cytokines can be classified in various ways, such as by their function, structure, target cells, role within the immune system, or receptor families. In the context of endometriosis, where a chronic inflammatory component plays a significant role, the classification of cytokines into pro-inflammatory and anti-inflammatory categories is the most commonly used approach (Figure 1).

**Figure 2:**
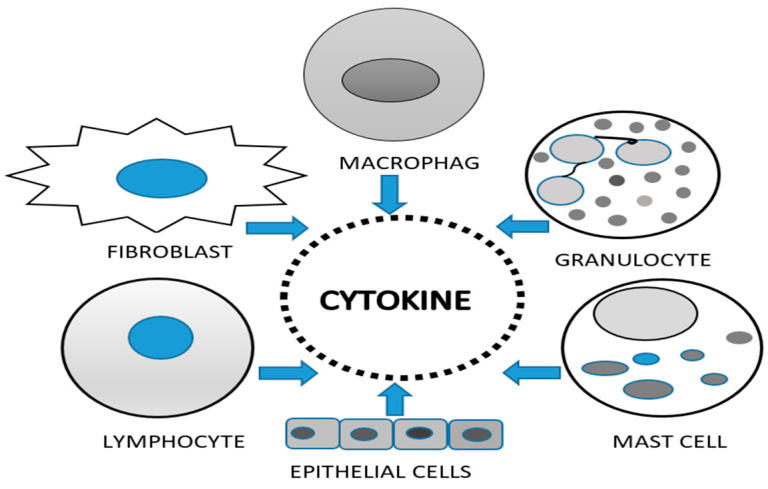
Essential cells in cytokines production.

Cytokines affect processes such as cell motility, adhesion, chemotaxis, and morphogenesis, potentially influencing or modulating the progression of endometriosis. They are found in blood plasma, endometrial tissue, follicular fluid, peritoneal fluid, or urine and are produced by many types of cells, with immune cells being the primary producers (Figure 2).

Genetic polymorphisms can influence cytokine expression levels and activity, thereby affecting disease manifestation and progression [8]. Cytokines have been studied as potential biomarkers alone and by several groups. Based on an extensive review of the literature, we selected the cytokines most commonly investigated in endometriosis studies for detailed analysis. Although findings across studies are heterogeneous, there is a general consensus that a combination of specific biomarkers, rather than a single marker, may enhance diagnostic accuracy for endometriosis. This review aims to analyze the associations between these key cytokines and the pathogenetic mechanisms underlying endometriosis, providing information on genetic factors influencing cytokine production.

## 2. Literature Review

### 2.1. Pro-Inflammatory Cytokines in the Diagnosis of Endometriosis

Pro-inflammatory cytokines are involved in the inflammatory response. In endometriosis, elevated levels of pro-inflammatory cytokines can promote the growth and survival of ectopic endometrial tissue, leading to tissue infiltration, pain, and chronic inflammation.

#### 2.1.1. Interleukin 1 (IL-1)

Interleukin-1 (IL-1) is a pro-inflammatory cytokine family secreted by activated peritoneal macrophages into peritoneal fluid, playing a key role in initiating inflammatory cascades involving cytokines (interleukin-6 (IL-6), interleukin-8 (IL-8)), B cells, antibodies, matrix metalloproteinases (MMPs), and prostaglandins [12,13]. This contributes to cell growth, differentiation, and angiogenesis. To add, IL-1 refers to the interleukin-1 family, which consists of several cytokines, primarily, interleukin-1α (IL-1α) and interleukin-1β (IL-1β). While both are pro-inflammatory cytokines that play roles in the immune response, they are distinct proteins with different functions and regulatory mechanisms. IL-1α is often associated with local inflammation and is secreted by various cells, including macrophages, whereas IL-1β is typically released by activated immune cells during systemic inflammatory responses [14]. Higher levels of IL-1 have been found in women with endometriosis. While IL-1 generally regulates inflammation, excessive secretion may cause tissue damage and chronic inflammation; however, self-regulatory mechanisms within the IL-1 family help to mitigate this [14,15]. Meanwhile, one study indicates that IL-1α may have significant potential as a biomarker for diagnosing endometriosis. A study reported that cervico-vaginal fluid levels of IL-1α were substantially higher in women with endometriosis compared to controls. The optimal threshold for IL-1α was identified at 105 pg/mL, achieving a sensitivity and specificity of 100%. This suggests that IL-1α could be an effective non-invasive diagnostic marker for endometriosis [16].

Genetic polymorphisms in the *IL1* gene cluster can influence the expression levels of IL-1α and IL-1β, respectively [16,17]. Certain alleles of these polymorphisms are associated with altered cytokine production, potentially contributing to the inflammatory environment in endometriosis [16,17]. Studies have shown that women carrying specific IL1A and IL1B alleles may have an increased risk of developing endometriosis due to higher IL-1 production [17,18]. However, findings are inconsistent across different populations, and further research is needed to clarify these associations.

#### 2.1.2. Interleukin 2 (IL-2)

Interleukin 2 (IL-2) is a cytokine that promotes the proliferation and differentiation of B and T cells, activates and stimulates the growth of nonspecific cytolytic effector cells like natural killer (NK) cells and lymphokine-activated killers (LAKs), and plays a role in activating monocytes and macrophages. However, the role of IL-2 in the development of endometriosis is not well understood, as research findings are inconsistent [14,19]. Hsu et al. reported lower levels of IL-2 in both peripheral blood and peritoneal fluid, with a more significant decrease in advanced stages of the disease. However, they found no differences in IL-2 and interleukin-10 (IL-10) messenger RNA (mRNA) levels between the endometriosis patients and the control group [20]. In contrast, Gogacz et al. found elevated IL-2 levels in the peritoneal fluid of patients with endometriosis who also experienced infertility [21]. Unfortunately, its diagnostic performance (sensitivity and specificity) has not been thoroughly established in large-scale studies.

The rs2069762 polymorphism has been significantly associated with an increased risk of developing endometriosis. This polymorphism, located in the promoter region of the *IL2* gene, may alter IL-2 expression, impacting immune regulation and contributing to the onset and progression of the disease [22].

#### 2.1.3. Interleukin 6 (IL-6)

IL-6 is a pro-inflammatory cytokine secreted by macrophages and plays a crucial role in immune responses, particularly in the differentiation of B cells and activation of T cells [8,23,24]. Additionally, IL-6 has been found to inhibit the proliferation of endometrial cells [25]. Studies have shown that IL-6 levels are significantly elevated in both the serum and peritoneal fluid of women with endometriosis compared to controls [26,27,28,29,30]. For instance, Jiang et al. reported that serum IL-6 could diagnose endometriosis with a sensitivity of 90% and a specificity of 93.7%. Additionally, they observed a positive correlation between IL-6 levels in serum and peritoneal fluid and the severity of endometriosis. Significantly higher IL-6 levels were found in the advanced stages of the disease [31]. Bedaiwy and colleagues demonstrated that measuring serum IL-6 alongside peritoneal TNF-α accurately distinguishes between women with and without endometriosis [32]. IL-6 has been the most extensively studied biomarker and was included in a meta-analysis conducted by Nisenblat et al. An analysis of three studies revealed an average sensitivity of 63% (95% CI 52–75%) and specificity of 69% (95% CI 57–82%) for a cut-off value of >1.90–2.00 pg/mL. However, there were insufficient data for meta-analysis for other cut-off values [33]. Additionally, IL-6 was evaluated along with other potential biomarkers. Mihalyi et al. assessed a panel of six potential serum markers (IL-6, IL-8, TNF-α, high-sensitivity C-reactive protein, Ca-125, and Ca-19.9) for diagnosing endometriosis. They achieved a sensitivity of 100% and a specificity of 84% for moderate-to-severe endometriosis, and a sensitivity of 87% and a specificity of 71% for minimal-to-mild endometriosis [34]. Vodolazkaia et al. examined the specificity and sensitivity of 28 biomarkers in plasma samples from women with and without endometriosis. They found elevated levels of pro-inflammatory markers, including TNF-α, IL-6, and IL-1β, in the control group rather than in those with endometriosis. This suggested that non-endometriotic pelvic pathology may contribute to the elevated marker levels in the control group. As a result, the inappropriate selection of controls compromised the ability to differentiate between patients with endometriosis and those with other forms of pelvic inflammation and pain. This indicates that pro-inflammatory cytokines may not be reliable non-invasive biomarkers for endometriosis unless appropriate control subjects are used [35].

*IL6* gene polymorphisms have been extensively studied in relation to endometriosis. Systematic review and meta-analysis revealed that neither the GC + CC genotype versus GG genotype for rs1800795 nor the GC + GG genotype versus CC genotype for rs1800796 showed a significant association with the risk of endometriosis [36].

#### 2.1.4. Interleukin 15 (IL-15)

IL-15 is reported to be produced by a wide variety of cells and tissues, including epithelial cell lines, monocytes, macrophages, and decidual and endometrial tissues. It is a cytokine that promotes the growth of several T-cell types, such as CD4+, γδ, CD8+, and memory T cells. It is also involved in T-cell chemotaxis and interacts with NK cell growth and TNF-α, as well as with other key factors of immune system. Additionally, IL-15 has been shown to function as an angiogenic factor in in vivo models [37]. The involvement of IL-15 in angiogenesis and the proliferation of human endometrial endothelial cells has been previously documented, suggesting a potential role for IL-15 in the development of endometriosis [38,39]. Studies have reported elevated levels of IL-15 in the peritoneal fluid and ectopic endometrium of women with endometriosis [40,41]. However, research by Lin and colleagues found that IL-15 levels were decreased in peritoneal fluid during the advanced stages of endometriosis [42]. There is also a lack of specific sensitivity and specificity values for IL-15 in the context of diagnosing endometriosis, either alone or in combination with other biomarkers. Overall, more comprehensive studies are needed to clarify its role in endometriosis diagnostics.

Currently, there is no reliable information on the influence of *IL15* gene polymorphisms on cytokine levels in endometriosis.

#### 2.1.5. Interleukin 16 (IL-16)

IL-16, also known as lymphocyte chemoattractant factor, is a polypeptide pro-inflammatory cytokine that plays a crucial role in immune and inflammatory responses, including the development of endometriosis. It is produced by a variety of cell types previously found in association with complex disorders. IL-16 is now recognized as essential in regulating cellular functions. IL-16 has been shown to stimulate peripheral blood mononuclear cells to produce pro-inflammatory cytokines, including IL-6, IL-15, TNF-α, and IL-1β. Although it is clear that IL-16 acts as an inflammatory mediator, the exact mechanisms through which it operates remain under investigation [43,44]. In a study examining the role of IL-16 in endometriosis, researchers found that IL-16 concentrations in the peritoneal fluid of women with advanced endometriosis (stages III/IV) were significantly elevated (330 pg/mL) compared to those without endometriosis (229 pg/mL), with a statistical significance of *p* = 0.0016. Additionally, peripheral blood mononuclear cells (PBMCs) cultured with recombinant human IL-16 (rhIL-16) produced higher levels of IL-6, TNF-α, and IL-1β, with increases of 1.17, 1.33, and 1.54-fold, respectively, compared to the control. The findings suggest that IL-16 in the peritoneal fluid may contribute to the pathogenesis of endometriosis by initiating or maintaining inflammatory responses within the peritoneal cavity [45].

The rs4778889 polymorphism in the *IL16* gene promoter region was found to be significantly associated with the risk of endometriosis in the Nigerian population. The study demonstrated that individuals with the C allele of rs4778889 exhibited increased susceptibility to endometriosis, potentially due to its role in altering IL-16 expression levels and contributing to the inflammatory environment characteristic of the disease [46].

#### 2.1.6. Interleukin 17 (IL-17)

The IL-17 family is a group of pro-inflammatory cytokines produced by Th cells in response to interleukin 23 (IL-23) stimulation [47]. This family consists of six structurally similar members: IL-17A (often referred to as IL-17), IL-17B, IL-17C, IL-17D, IL-17E (also known as IL-25), and IL-17F. Increasing evidence highlights the critical role of IL-17 in the pathogenesis of endometriosis [8]. Studies show that IL-17 levels are significantly elevated in the serum and peritoneal fluid of women with endometriosis compared to controls [48,49,50]. Zhang et al. noted that women with minimal or mild endometriosis had higher IL-17 levels in peritoneal fluid than those with moderate or severe endometriosis, especially when endometriosis was accompanied by infertility. However, no correlation was found between IL-17 concentrations in peritoneal fluid and the menstrual cycle phase in either group [51]. Conversely, some studies reported no significant differences in IL-17 levels in serum and peritoneal fluid between women with and those without endometriosis [52,53]. Overall, IL-17 appears to be a promising target for further research; however, conclusive meta-analyses specifically detailing its sensitivity and specificity in diagnosing endometriosis have not yet been prominently published. This area remains a potential focus for future studies to better define the diagnostic capabilities of IL-17 in endometriosis.

Currently, there is no reliable information regarding *IL17* gene polymorphisms in cases of endometriosis.

#### 2.1.7. Interleukin 18 (IL-18)

IL-18 plays a crucial role in regulating the production of TNF-α and IL-8, functions as a strong angiogenic factor, and modulates the expression of adhesion molecule 1 via nuclear factor kappa B (NF-κB), potentially enhancing the production of MMPs. IL-18 is a major regulator of the immune response process in a wide range of cells that decreases in both eutopic and ectopic endometrium in endometriosis [2,54]. Arici et al. aimed to investigate the levels of IL-18 in the peritoneal fluid of women with and without endometriosis. The study involved 50 women with untreated endometriosis, eight women on GnRH agonists for endometriosis, and 18 control women with normal pelvic anatomy. The results showed that IL-18 levels were significantly higher in women with untreated endometriosis (91.1 pg/mL) compared to controls (59.4 pg/mL). Notably, women with superficial and deep peritoneal implants had elevated IL-18 levels, while those with endometriomas showed lower levels. Additionally, women with early-stage endometriosis had higher IL-18 levels than controls, whereas those with advanced stages did not. Furthermore, IL-18 levels were elevated during the luteal phase compared to the follicular phase, but they did not differentiate between women with pelvic pain and those with infertility. The findings suggest that elevated IL-18 in peritoneal fluid is associated with minimal-to-mild stages of endometriosis [55]. However, another study showed that no statistically significant differences were detected in IL-18 levels in either serum or peritoneal fluid samples [56]. One study aimed to measure IL-18 concentrations in the serum and peritoneal fluid of infertile women with endometriosis. A total of 34 infertile women with minimal or mild endometriosis and 22 fertile controls were analyzed. No significant differences were found in IL-18 levels between the two groups in either serum or peritoneal fluid. However, a positive correlation was observed between serum and peritoneal IL-18 levels in the endometriosis patients (r = 0.794, *p* = 0.0001). The findings did not support the hypothesis that IL-18 levels are associated with infertility in women with minimal or mild endometriosis [57].

The rs1946518 polymorphism in the *IL18* gene can affect IL-18 expression. Some studies have suggested that this polymorphism may be associated with endometriosis risk, possibly through the modulation of inflammatory and immune responses [58].

#### 2.1.8. Interleukin 33 (IL-33)

IL-33 is an alarmin from the IL-1 family that mainly interacts with both the innate and adaptive immune systems, playing a role in infectious and chronic inflammatory conditions [8]. Miller et al. discovered that patients with stage III and IV endometriosis produce significantly higher levels of IL-33 in their endometriotic lesions compared to the eutopic endometrial tissues of healthy and fertile individuals. This increased IL-33 can be detected in the peripheral blood and peritoneal fluid of endometriosis patients [59]. Additionally, another study reported elevated IL-33 levels in the peritoneal fluid of women with endometriosis, which correlated with the severity of the disease [60]. Conversely, Jaeger-Lansky et al. found no significant differences in IL-33 levels in the peritoneal fluid between women with and those without endometriosis [61]. Combining IL-33 with other established biomarkers may enhance diagnostic accuracy, but more research is needed.

Research into *IL33* gene polymorphisms in endometriosis patients may help clarify their role in cytokine regulation and disease severity. A study by Albertsen et al. unveiled the rs10975519 polymorphism in the *IL33* gene as an independent factor for endometriosis [62].

#### 2.1.9. Monocyte Chemotactic Protein 1 (MCP-1)

MCP-1 promotes endometriosis by driving monocytes to migrate into the peritoneal cavity, where they differentiate into macrophages, fueling local inflammation. Studies have shown that peritoneal macrophages are more abundant in women with endometriosis compared to controls [63]. Research indicates that levels of MCP-1 significantly rise during the luteal phase [64]. However, a threshold of 100 pg/mL for MCP-1 achieved only 65% sensitivity and 61% specificity. Additionally, the stage of endometriosis may influence MCP-1’s diagnostic value: one study reported higher levels in early disease stages [65], while another found elevated levels in more advanced stages [66].

There is currently no reliable information available on the influence of *MCP1* gene polymorphisms on cytokine levels in endometriosis.

#### 2.1.10. Tumor Necrosis Factor Alpha (TNF-α)

TNF is a pro-inflammatory cytokine primarily produced by activated macrophages, T lymphocytes, and NK cells. TNF-α plays a significant role in the formation and development of endometriosis implants [12]. The marker is responsible for increasing vascular permeability and activating inflammatory factors in the peritoneal cavity, which can worsen peritonitis [2]. The severity of endometriosis is strongly influenced by the serum levels of TNF-α [67].

Bedaiwy et al. investigated the prognostic value of six cytokines (IL-1β, IL-6, IL-8, IL-12, IL-13, and TNF-α) for the diagnosis of endometriosis. Cytokine concentrations were measured in both blood and peritoneal fluid. Laparoscopic surgeries were performed on women with chronic pain, for tubal ligation, or for tubal reconstruction after prior sterilization. TNF-α in peritoneal fluid showed 100% sensitivity and 89% specificity for diagnosing endometriosis [32].

An evaluation of the diagnostic value of TNF-α soluble transmembrane receptors (sTNFR1 and sTNFR2) in endometriosis revealed that the levels of both receptors were significantly higher in the early stages of endometriosis, as classified by rASRM system, compared to the control group (sTNFR1, *p* < 0.01; sTNFR2, *p* < 0.04). However, in the advanced stages, the levels of both receptors were not significantly different from those observed in the control group. In this study, the test group comprised patients with laparoscopically confirmed endometriosis, while the control group included patients who underwent laparoscopy for pelvic pain or infertility but showed no visually detectable endometriosis. All surgeries were performed during the proliferative or secretory phases, and blood tests for sTNFR1 and sTNFR2 were conducted prior to surgical interventions [68].

Comparing data from three studies (256 patients), the sensitivity of TNF-α for diagnosing endometriosis ranged from 68% to 89%, while the specificity ranged from 35% to 87% [33]. A meta-analysis of these studies was not feasible because each study reported different positive threshold values (TNF-α levels showing a significant difference between patients with and without endometriosis). However, in the same analysis, a review of eight other studies (633 patients) that examined TNF-α levels in the blood, regardless of the stage of endometriosis, found no statistically significant difference between patients with endometriosis and healthy controls.

Polymorphisms in the *TNF* gene (rs361525, rs1800629, rs1799724, rs1800630, and rs1799964) were evaluated, with the analysis revealing that only the rs1799964 variant was significantly associated with an increased risk of endometriosis [36].

#### 2.1.11. Interferon Gamma (IFNγ)

IFNγ is a glycoprotein and cytokine that plays a crucial role in both innate and adaptive immunity. There are three types of IFN in the human body: type I includes interferon-alpha (IFNα), interferon-beta (IFNβ), and interferon-epsilon (IFNε) in some placental mammals and interferon-delta (IFNδ) and interferon-tau (IFNτ) in non-primate and non-rodent placental mammals; type II includes interferon gamma (IFNγ); and type III includes interferon lambda (IFNλ) [69].

While the roles of interferons (IFNs) in endometriosis have not been thoroughly investigated, existing research suggests that IFN signaling is impaired in both ectopic lesions and the eutopic endometrium of patients with endometriosis. This disruption may play a role in the disease’s development and its associated symptoms [69,70].

IFNγ is primarily produced by activated T and NK cells, and it plays a crucial role in modulating the cellular immune response. This includes contributing to macrophage activation and T-cell development. Additionally, IFNγ is an important cytokine that interacts with IL-2 to help regulate the balance between Th1 and Th2 cells [14,71].

One of the first studies measuring the concentration of this cytokine in peritoneal fluid was published in 1996 [72]. The study included patients who were evaluated for infertility. They were divided into two groups: the first group consisted of women with confirmed endometriosis during surgery, while the second group included infertile women without this condition. In all patients with endometriosis, significantly elevated levels of IFNγ were found compared to those without the disease. Patients with stage III and IV endometriosis, as determined by the rASRM (American Society of Reproductive Medicine), were treated for six months with medications from the gonadotropin-releasing hormone (GnRH) agonist group, followed by “second-look” laparoscopy to reassess the cytokines in peritoneal fluid. IFNγ concentrations in peritoneal fluid were significantly reduced. Hsu et al. reported similar findings, supporting this observation [20]. In contrast, Podgaec et al. observed elevated IFNγ levels in the peritoneal fluid near lesions in endometriosis patients, with no significant differences in peripheral blood [73].

Evidence suggests that the sensitivity and specificity of measuring IFNγ in blood plasma for diagnosing endometriosis at any stage are 0.68 and 0.63, respectively [34]. However, studies examining this cytokine in the blood and correlating its concentration changes with endometriosis are highly heterogeneous. A summary of five studies found no statistically significant differences in the analysis of IFNγ concentrations in blood plasma between patients with endometriosis and those without the disease [33].

The summary of the results of two studies shows that the IFNγ a13 allele was positively associated with the risk of endometriosis [36].

### 2.2. The Role of Anti-Inflammatory Cytokines

Anti-inflammatory cytokines play an important role in modulating the inflammatory processes in endometriosis. Their primary function is to counteract pro-inflammatory cytokines, reduce inflammation, and maintain tissue homeostasis. The key anti-inflammatory cytokines and their role are outlined below.

#### 2.2.1. Interleukin 4 (IL-4)

IL-4 is an anti-inflammatory cytokine produced by CD4+ T cells that plays various biological roles [74]. The main function of IL-4 is to help naive T cells differentiate after they encounter an antigen. Through this process, these T cells become helper T (Th) cells that primarily promote a Th2-type immune response [75,76]. In endometriosis, studies have repeatedly shown that disease progression is linked to Th2 immune activation [20,77,78]. IL-4 and IL-10 work together to counteract inflammatory cytokines like interferon gamma (IFNγ), IL-2, interleukin-3 (IL-3), and tumor necrosis factor alpha (TNF-α). These cytokines promote a Th2 response, which reduces the cytotoxic activity that would normally help eliminate ectopic endometrial tissue [79,80]. In endometriosis implants, IL-4 encourages eotaxin secretion, leading to angiogenesis and the progression of lesions. At the same time, IL-4’s anti-inflammatory properties can reduce the formation of adhesions in the peritoneal cavity [20]. Studies have found that cells producing IL-4 are present in ectopic endometrial tissue, and that IL-4 stimulates the activity of endometrial stromal cells [81,82]. Drosdzol-Cop et al. found that both serum and peritoneal fluid levels of IL-4 were significantly higher in young individuals with endometriosis [83]. Furthermore, recent studies have shown that serum IL-4 levels are notably elevated in patients with endometriosis compared to controls, suggesting that IL-4 could serve as a valuable biomarker for diagnosing the condition [18,84]. However, there are no well-defined studies specifically measuring its sensitivity and specificity as a diagnostic marker.

A meta-analysis found no significant association of *IL4* genetic polymorphism rs2243250 with endometriosis [36].

#### 2.2.2. Interleukin-1 Receptor Antagonist (IL-1RA)

The interleukin-1 receptor antagonist (IL-1RA) is the anti-inflammatory cytokine and part of the extensive IL-1 family, which includes 11 members that display both pro-inflammatory (such as IL-1, interleukin-18 (IL-18), and interleukin-36 (IL-36)) and anti-inflammatory (such as IL-1RA and interleukin-37 (IL-37)) properties. Research by Kondera-Anasz et al. demonstrated that levels of IL-1RA were significantly elevated in the serum and peritoneal fluid of patients with endometriosis [17]. Similarly, Malutan et al. found that serum levels of IL-1RA were significantly higher in women with endometriosis compared to those without the condition [18]. However, Zhang et al. reported a decrease in IL-1RA levels in the peritoneal fluid of endometriosis patients, particularly in those experiencing dysmenorrhea [85]. There is a lack of sufficient information about sensitivity and specificity in publications.

Genetic variations in the *IL1RN* gene, which encodes IL-1RA, have been studied in the context of endometriosis. The *IL1RN* variable number tandem repeat (VNTR) polymorphism has been associated with altered levels of IL-1RA and susceptibility to endometriosis. The presence of certain alleles may lead to increased production of IL-1RA, which could modulate the inflammatory response in the peritoneal cavity [86].

#### 2.2.3. Interleukin 10 (IL-10)

IL-10 is an anti-inflammatory cytokine that suppresses the production of various cytokines, such as IFNγ, IL-2, IL-3, TNF, and granulocyte-macrophage colony-stimulating factor (GM-CSF), and is typically produced by activated macrophages and Th cells [8]. Numerous studies have shown that IL-10 levels in both serum [19,47,48,49] and peritoneal fluid [48,73,77,87,88,89,90,91,92] are significantly increased in women with endometriosis compared to controls. On the other hand, Ahn et al. found that an imbalance in the local levels of this cytokine promotes the implantation of endometrial fragments into the peritoneal cavity [93]. Research using a mouse model of surgically induced endometriosis led the authors to conclude that changes in IL-10 concentration are associated with the growth of ectopic endometrial tissue [94]. The sensitivity and specificity of IL-10 as a standalone diagnostic marker for endometriosis are generally considered limited. The combination of IL-10 with other markers could improve diagnostic accuracy, but further research is needed to validate its effectiveness in clinical settings.

Polymorphisms within the *IL10* gene exhibit varying associations with endometriosis. While rs1800471 and rs1800896 show no significant correlation with an elevated risk of endometriosis, the rs1800872 variant has been linked to an increased risk of endometriosis [36].

#### 2.2.4. Interleukin 13 (IL-13)

IL-13 is known to play a role in Th2 responses and is commonly linked to allergic inflammation. It is released by T cells, mast cells, and eosinophils, and it can stimulate collagen production in fibroblasts and the formation of transforming growth factor beta (TGF-β) [95]. Chegini et al. reported a notable increase in IL-13 levels in the peritoneal fluid of patients with endometriosis, although this increase was not statistically significant [40]. Wang et al. found that IL-13 levels in the peritoneal fluid of endometriosis patients with infertility were significantly higher than in healthy controls [96]. However, some studies showed that patients with endometriosis had much lower levels of IL-13 in their serum and peritoneal fluid compared to the control group [97,98]. Inconsistencies in the findings of different studies may be due to variability in the clinical phase of the disease and the stage of a patient’s menstrual cycle. More research is needed to clarify its diagnostic value when used alone or with other biomarkers.

There is limited evidence connecting *IL13* gene polymorphisms to the disease, and more research is needed.

#### 2.2.5. Interleukin 37 (IL-37)

IL-37 is part of the IL-1 family and plays a role in suppressing the release of pro-inflammatory cytokines, acting as a natural regulator of innate inflammatory responses [99]. It has been found in both ectopic and eutopic endometrial tissues of women with endometriosis, indicating its potential role in inflammatory processes [100]. Several studies have reported that levels of IL-37 are significantly elevated in the peripheral blood and peritoneal fluid of women with endometriosis [31,101]. More research is needed to clarify its diagnostic value when used alone or with other biomarkers.

*IL37* gene polymorphisms have been studied in relation to other inflammatory conditions but not extensively in endometriosis [8]. Further research could explore whether genetic variations in *IL37* influence cytokine levels and impact the disease.

### 2.3. The Role of Dual-Action Cytokines in Endometriosis

Some cytokines exhibit dual roles, functioning as both pro-inflammatory and anti-inflammatory depending on the context, tissue environment, and type of immune response activated. In the case of endometriosis, these dual-action cytokines can contribute to the complex interplay between inflammation and immune tolerance that characterizes the condition. The following sections focus on the most important cytokines with a dual role.

#### 2.3.1. Interleukin 25 (IL-25) and Thymic Stromal Lymphopoietin (TSLP)

The endogenous cytokines IL-25 and TSLP play roles in the maturation of Th2 cells through dendritic cell activation. Additionally, IL-25 and TSLP stimulate innate immune cells, including type 2 innate lymphoid cells, to activate and produce IL-13 [1]. These cytokines are key regulators in allergic diseases, such as asthma, which develop in a Th2 cytokine environment [102]. Increasing evidence suggests that interleukin-33 (IL-33) and TSLP levels are elevated in the peripheral blood, endometriotic endometrium, and peritoneal fluid of individuals with endometriosis [59,60,103,104]. One study found higher IL-25 levels in the peritoneal fluid of endometriosis patients, though these levels did not correlate with disease stage [49]. Unfortunately, there is limited specific information regarding the sensitivity and specificity of IL-25 and TSLP in diagnosing endometriosis.

There is currently no reliable information available on the influence of *IL25* and *TSLP* gene polymorphisms on cytokine levels in endometriosis.

#### 2.3.2. Interleukin 27 (IL-27)

IL-27 is a proinflammatory cytokine that induces IL-10 production by Th17 cells in the endometriotic environment [1]. One article showed that interleukins IL-2 and IL-27 work together to enhance the growth and invasion of endometriotic stromal cells by regulating the balance between IFNγ and IL-10 in the context of endometriosis. This suggests a potential mechanism by which these cytokines contribute to the pathophysiology of endometriosis and highlights their importance in the inflammatory environment associated with this condition [105]. There appears to be a lack of studies specifically measuring IL-27 concentrations in serum and peritoneal fluid among patients with endometriosis. Most current research focuses on various other cytokines and their roles in endometriosis, but IL-27 has not been widely investigated as a potential diagnostic biomarker.

The role of *IL27* gene polymorphisms in endometriosis remains insufficiently explored.

#### 2.3.3. Transforming Growth Factor Beta (TGF-β)

TGF-β is a growth factor involved in multiple pathways, including the regulation of cell differentiation, proliferation, angiogenesis, and immune responses. Notably, disruptions in TGF-β signaling have been linked to various disorders [14]. Researchers suggest that TGF-β in endometriosis may be responsible for inhibiting NK cell activity in the peritoneal fluid [106]. This theory aligns with the concept of retrograde menstruation, suggesting that impaired NK cell function allows endometrial cells to survive and implant within pelvic structures. Additionally, reduced NK cell activity in the peritoneal fluid of women with endometriosis further supports this idea [107,108,109]. Numerous studies have found significantly higher levels of TGF-β in the serum and peritoneal fluid of patients with endometriosis compared to healthy controls [48,87,110]. However, comprehensive studies quantifying its sensitivity and specificity specifically for diagnosing endometriosis are lacking.

A recent meta-analysis indicated that the *TGFB* polymorphism (rs1800469) has no association with the risk of endometriosis [36].

#### 2.3.4. Vascular Endothelial Growth Factor (VEGF)

VEGF is a heparin-binding protein essential for the normal development and maintenance of blood vessels and the lymphatic system. Endometriosis is marked by increased blood vessel formation both within and around the ectopic tissue, leading to the speculation that the levels of the potent angiogenic growth factor VEGF in peritoneal fluid could be highly significant clinically [14]. Research indicates that women with endometriosis have higher VEGF levels in their peritoneal fluid compared to healthy women. The main source of VEGF comes from peritoneal fluid macrophages. Additionally, using anti-VEGF antibodies can prevent the increased growth of endothelial cells caused by substances from these macrophages. Therefore, we can conclude that the expression of VEGF in endometriosis is influenced by estradiol and progesterone [111].

Studies indicate that patients with endometriosis have significantly higher VEGF levels, with notable associations between VEGF levels and disease stage. In their examination of VEGF concentrations in blood and peritoneal fluid, Foda and colleagues also observed that the sensitivity, specificity, and diagnostic accuracy of blood VEGF testing were lower than those of IL-6, being 91.67%, 76.67%, and 86.67%, respectively [112]. In a study conducted by Mohamed et al., results indicated a significant difference in serum levels of both Ca-125 and VEGF-A between patients with advanced endometriosis and controls before and after surgery (*p* < 0.001). VEGF-A demonstrated higher sensitivity (93.3%), specificity (96.7%), and accuracy (95.0%) in distinguishing endometriosis patients from controls compared to Ca-125, which showed a sensitivity of 70.0%, specificity of 90.0%, and accuracy of 85.0%. Furthermore, the percentage decrease in VEGF-A levels post-surgery (45.9%) was significantly greater than that of Ca-125 (25.8%) (*p* < 0.001). This suggests that VEGF-A may be a more reliable biomarker for diagnosing and monitoring advanced endometriosis than Ca-125 [113].

Despite extensive clinical data, information on genetic factors associated with VEGF secretion in endometriosis is scarce. One study identified the *VEGF* +405G>C polymorphism as a potential risk factor for endometriosis, particularly in cases involving endometrioid cysts exceeding 3 cm in size [114].

A summary of cytokine properties relevant to endometriosis diagnostics and pathogenesis is provided in Table 1.

### 2.4. Limitations of Using Cytokines in the Diagnosis of Endometriosis

Despite the increasing scientific understanding of the role of cytokines in the pathogenesis and diagnosis of endometriosis, their practical and clinical application remains challenging. First, cytokines exhibit a lack of specificity as potential biomarkers. Cytokines are involved in a broad range of inflammatory diseases, making it difficult to use them as specific biomarkers for endometriosis. Elevated levels of cytokines such as IL-6 and TNF-α can also be observed in other conditions like pelvic inflammatory disease, infections, and autoimmune disorders. This lack of specificity reduces the diagnostic accuracy of cytokines when used alone.

Another contributing factor is the variability of cytokine profiles. Cytokine levels can vary greatly between individuals and even within the same individual at different stages of endometriosis. Factors such as disease severity, lesion location, hormonal fluctuations, and even sample collection methods can influence cytokine levels, leading to inconsistent results. This variability challenges the reliability of cytokines as diagnostic markers.

Another issue is the complex immune response. The immune response in endometriosis is highly dynamic, with cytokines often functioning in a context-dependent manner. Some cytokines can act as both pro-inflammatory and anti-inflammatory mediators, depending on the immune environment. For example, IL-6 can promote inflammation in one setting while also inducing the release of the anti-inflammatory cytokine IL-10 in another. This duality complicates the interpretation of cytokine levels and their role in disease progression.

Differences between local and systemic measurements presents another challenge. While cytokines are involved in the local immune response within the peritoneal cavity, blood-based assays may not accurately reflect the cytokine profiles at the site of endometriotic lesions. Consequently, diagnostic approaches relying solely on peripheral blood samples may miss important local immune responses, further complicating the use of cytokines as biomarkers.

The lack of standardized cut-offs should be considered. Despite various studies identifying altered cytokine levels in endometriosis, no universally accepted thresholds or cut-off values exist to distinguish between healthy individuals and those with endometriosis. The absence of clear diagnostic criteria limits the clinical applicability of cytokines in routine practice.

Finally, the significance of cytokine gene polymorphisms should be considered. Cytokine gene polymorphisms may be significant for the diagnosis of endometriosis, as they can influence immune and inflammatory responses. Specific polymorphisms in genes such as *TNF*, *IL6*, and *IL10*, which are involved in inflammation and immune reactions, may increase the risk of the disease. However, their application in diagnostics is still limited, as their significance can vary between individuals and depends on the severity of the disease. Further research is needed to reliably use genetic biomarkers in diagnostics.

To enhance the diagnostic utility of cytokines in endometriosis, a panel of cytokines offers a broader perspective on the immune response, thereby improving diagnostic accuracy. It offers a more holistic and accurate understanding of the immune environment in endometriosis, improving diagnostic reliability and clinical utility.

## 3. Conclusions

Cytokines are molecules that play an important role in immune responses and inflammatory processes. When evaluating their diagnostic value for endometriosis, it is observed that some cytokines may show potential as biomarkers, but their practical use is limited due to various challenges. The heterogeneity of existing studies, including variations in study populations and methodologies, contributes to inconsistent findings, which complicates the assessment of their diagnostic utility. Robust meta-analyses and the inclusion of appropriately matched control groups are essential to address these limitations. In summary, cytokines have the potential to be useful diagnostic markers for endometriosis, but their diagnostic value is currently limited, and further research is needed to evaluate their accuracy and reliability.

## Figures and Tables

**Table 1:** Summary of the origin, function, expression, and regulation of various cytokines in endometriosis.

Cytokine	Origin	Function	Expression in Endometriosis	Regulation (Genetic Polymorphisms)
Interleukin-1 (IL-1)	Peritoneal macrophages	Pro-inflammatory Promotes cell growth, differentiation, and angiogenesis	↑ Cervico-vaginal, peritoneal fluid, serum, and plasma	rs1143634 in *IL1:* no significant association with endometriosis
Interleukin-2 (IL-2)	T and NK cells	Pro-inflammatory Enhances T-cell proliferation and NK cells	Mixed findings	rs2069762 in *IL2*: associated with predisposition of endometriosis
Interleukin-6 (IL-6)	Macrophages	Pro-inflammatory Involved in B-cell differentiation and T-cell activation	↑ Serum and peritoneal fluid	rs1800795 and rs1800796 in *IL6:* no significant association with endometriosis
Interleukin-15 (IL-15)	Epithelial cells and macrophages	Pro-inflammatory Promotes growth of T-cell types; involved in chemotaxis and angiogenesis	↑ Peritoneal fluid and ectopic endometrium (some studies show decreased levels in advanced stages)	No reliable information
Interleukin-16 (IL-16)	Immune (T cells, eosinophils, neutrophils, and dendritic cells) and non-immune (fibroblast, epithelial, and neuronal) cells	Pro-inflammatory; Crucial role in immune and inflammatory responses	↑ Peritoneal fluid in advanced stages	rs4778889 in *IL16*: associated with higher risk of endometriosis
Interleukin-17 (IL-17)	Th17 cells	Pro-inflammatory Involved in immune responses	Mixed findings	No reliable information
Interleukin-18 (IL-18)	Hematopoietic and non-hematopoietic cells, including monocytes, macrophages, keratinocytes, and mesenchymal cells	Pro-inflammatory Modulates expression of adhesion molecules; promotes angiogenesis	Mixed findings	rs1946518 in *IL18:* associated with higher risk of endometriosis
Interleukin-33 (IL-33)	Endothelial cells, fibroblasts, and macrophages	Pro-inflammatory Interacts with innate and adaptive immune systems	Mixed findings	rs10975519 in *IL33:* associated with higher risk of endometriosis
Monocyte Chemotactic Protein-1 (MCP-1)	Produced by monocytes/macrophages	Pro-inflammatory Promotes monocyte migration and differentiation into macrophages	↑ Peritoneal fluid	No reliable information
Tumor Necrosis Factor Alpha (TNF-α)	Produced by activated macrophages, T lymphocytes, and NK cells	Pro-inflammatory Involved in inflammatory responses	↑ Peritoneal fluid	rs1799964 in *TNF*: associated with higher risk of endometriosis
Interferon Gamma (IFNγ)	Produced by T cells and NK cells	Pro-inflammatory Key role in innate and adaptive immunity	Mixed findings	IFNγ a13 allele: associated with higher risk of endometriosis
Interleukin-4 (IL-4)	CD4+ T cells	Anti-inflammatory Promotes Th2 immune response; aids in T-cell differentiation	↑ Serum and peritoneal fluid	rs2243250 in *IL4:* no significant association with endometriosis
Interleukin-1 Receptor Antagonist (IL-1RA)	Hepatic cells, immune cells, epithelial cells, and adipocytes	Anti-inflammatory Regulates IL-1 activity by acting as a receptor antagonist	↑ Serum and peritoneal fluid (some studies show decreased levels in peritoneal fluid)	VNTR in *IL1RN*: associated with higher risk of endometriosis
Interleukin-10 (IL-10)	Macrophages and Th cells	Anti-inflammatory Suppresses production of various cytokines	↑ Serum and peritoneal fluid	rs1800872 in *IL10:* associated with higher risk of endometriosis
Interleukin-13 (IL-13)	T cells, mast cells, and eosinophils	Anti-inflammatory Promotes Th2 responses; stimulates collagen production; induces TGF-β formation	Mixed findings	No reliable information
Interleukin-37 (IL-37)	Monocytes and macrophages	Anti-inflammatory Suppresses release of pro-inflammatory cytokines	↑ Serum and plasma and peritoneal fluid	No reliable information
Interleukin-25 (IL-25)	T cells, NK cells, and dendritic cells	Pro- and anti-inflammatory Promotes Th2 responses	↑ Peritoneal fluid	No reliable information
Thymic Stromal Lymphopoietin (TSLP)	Epithelial cells, fibroblasts, stromal cells, and keratinocytes	Pro-inflammatory Promotes Th2 responses and inflammation	↑ Serum and plasma, endometrium, and peritoneal fluid	No reliable information
Interleukin-27 (IL-27)	Monocytes, macrophages, and dendritic cells	Pro- and anti-inflammatory Enhances growth and invasion of endometriotic stromal cells; regulates balance between IFNγ and IL-10	Not widely investigated	No reliable information
Transforming Growth Factor Beta (TGF-β)	T cells, platelets	Pro- and anti-inflammatory Regulates cell differentiation, proliferation, and angiogenesis; inhibits NK cell activity	↑ Serum and peritoneal fluid	rs1800469 in *TGFB*: no significant association with endometriosis
Vascular Endothelial Growth Factor (VEGF)	Macrophages, endothelial cells, fibroblasts, and platelets	Pro- and anti-inflammatory Promotes angiogenesis; essential for blood vessel development	↑ Serum and peritoneal fluid	+405G>C in *VEGF:* associated with higher risk of endometriosis

↑—Elevated or increased levels.

## Abbreviations

Ca-125	Cancer antigen 125
Ca-19.9	Cancer antigen 19.9
IL-1	Interleukin-1
IL-1α	Interleukin-1 alfa
IL-1β	Interleukin-1 beta
IL-2	Interleukin-2
IL-4	Interleukin-4
IL-6	Interleukin-6
IL-10	Interleukin-10
IL-13	Interleukin-13
IL-15	Interleukin-15
IL-16	Interleukin-16
IL-17	Interleukin-17
IL-18	Interleukin-18
IL-25	Interleukin-25
IL-27	Interleukin-27
IL-33	Interleukin-33
IL-37	Interleukin-37
IL-1RA	Interleukin-1 receptor antagonist
IFNα	Interferon alpha
IFNβ	Interferon beta
IFNε	Interferon epsilon
IFNδ	Interferon delta
IFNτ	Interferon tau
IFNγ	Interferon gamma
IFNλ	Interferon lambda
LAKs	lymphokine-activated killers
MCP-1	Monocyte chemotactic protein 1
mRNA	Messenger ribonucleic acid
miRNAs	Micro ribonucleic acid
MMPs	matrix metalloproteinases
NF-κB	nuclear factor kappa B
NK	Natural killer
PBMCs	Peripheral blood mononuclear cells
rASRM	American Society of Reproductive Medicine
rhIL-16	Recombinant human Interleukin-16
sTNFR	TNF-alpha soluble transmembrane receptors
Th1	T-helper 1
Th2	T-helper 2
TGF-β	Transforming growth factor beta
TNF-α	Tumor necrosis factor alpha
TSLP	Thymic stromal lymphopoietin
VEGF	Vascular endothelial growth factor
